# Effect of Water and Organic Pollutant in CO_2_/CH_4_ Separation Using Hydrophilic and Hydrophobic Composite Membranes

**DOI:** 10.3390/membranes10120405

**Published:** 2020-12-08

**Authors:** Clara Casado-Coterillo, Aurora Garea, Ángel Irabien

**Affiliations:** Department of Chemical and Biomolecular Engineering, Universidad de Cantabria, Av. Los Castros s/n, 39005 Santander, Spain; gareaa@unican.es (A.G.); irabienj@unican.es (Á.I.)

**Keywords:** composite membranes, CO_2_/CH_4_ separation, water and organic pollutants, hydrophilic/hydrophobic character, biogas upgrading, sustainable energy

## Abstract

Membrane technology is a simple and energy-conservative separation option that is considered to be a green alternative for CO_2_ capture processes. However, commercially available membranes still face challenges regarding water and chemical resistance. In this study, the effect of water and organic contaminants in the feed stream on the CO_2_/CH_4_ separation performance is evaluated as a function of the hydrophilic and permselective features of the top layer of the membrane. The membranes were a commercial hydrophobic membrane with a polydimethylsiloxane (PDMS) top layer (Sulzer Chemtech) and a hydrophilic flat composite membrane with a hydrophilic [emim][ac] ionic liquid–chitosan (IL–CS) thin layer on a commercial polyethersulfone (PES) support developed in our laboratory. Both membranes were immersed in NaOH 1M solutions and washed thoroughly before characterization. The CO_2_ permeance was similar for both NaOH-treated membranes in the whole range of feed concentration (up to 250 GPU). The presence of water vapor and organic impurities of the feed gas largely affects the gas permeance through the hydrophobic PDMS membrane, while the behavior of the hydrophilic IL–CS/PES membranes is scarcely affected. The effects of the interaction of the contaminants in the membrane selective layer are being further evaluated.

## 1. Introduction

Membrane gas separation of CO_2_ from synthesis gas, natural gas or biogas is a potential energy-efficient alternative to other separation techniques with potential for biogas upgrading as an alternative energy source from anaerobic digestion and landfills [1,2]. The availability of organic biomass could facilitate countries in meeting sustainable development goals related with creating and providing sustainable energy sources [3]. The typical components of biogas are mainly high added value as combustible methane and the major non-combustible component CO_2_, together with traces of impurities, such as H_2_S, water vapor, aromatics, chlorinated hydrocarbons, volatile organic compounds and siloxanes [4,5,6,7].

Separation of CO_2_ from methane is also important to use biogas as a natural gas substitute [3]. CO_2_/CH_4_ separation is particularly challenging due to the variability of the biogas composition, which depends on the source and seasonal conditions [7]. Both feed flow rate and feed concentrations change during the CO_2_/CH_4_ separation when the feed concentration of the valuable component is at least 65 mol % at a pressure in the range between 3.1 and 4.1 bar [8]. 

Biogas upgrading, thus, does not require high pressure and temperatures as other CO_2_/CH_4_ separations, such as natural gas separation. In 1989, a two-stage process involving an adsorption step to remove the impurities and a membrane unit for CO_2_ separation was first proposed for biogas’s upgrading [9]. Since then, several small landfills in Europe have set up pilot membrane biogas upgrading plants, but they are still not commercially available [3]. The correlation of theoretical and technological studies should emphasize feasibility based on laboratory-scale experiments for further development of biogas plants to large-scale projects, by improving stability and efficiency [10]. In this light, there remains significant scope for the development of better performing CO_2_ selective membranes to improve the separation power and the durability of materials [11].

There are different commercial polymeric membranes for CO_2_ separation processes made of different materials, basically polymers: Polaris^®^ ES Polaris^TM^ EN LUGAR DE Polaris^®^? (Membrane Technology Research, Inc. (Newark, CA, USA), MTR) [12], and Dupont/Air Liquid, Air Products & Chemicals Inc. (Allentown, PA, USA) [13], General Electric (Boston, MA, USA) [14], Honeywell (Charlotte, NC, USA) [15] and Evonik (Essen, Germany) [16] are also other major players in membrane technology. Their main advantages are their commercial availability or scale, low cost and easy reproducibility. The main drawbacks are the thermal, mechanical and chemical resistance, as well as the uncertainty of their performance regarding the presence of impurities such as water and organic vapors. A major challenge for developing effective gas separation membranes is overcoming the well-known permeability–selectivity trade-off for light bases in polymeric materials. Since popularized by Robeson in 2008, [17] this upper bound constituted the reference for characterizing advances in highly permselective membrane materials. Recently, deviations of the upper bound have been reported in mixed CO_2_/CH_4_ gas permeation experiments, for CO_2_/CH_4_ separation, for some of the mostly studied membranes in biogas upgrading, like cellulose acetate (CA) and polyimides [18], mixed matrix membranes (MMMs) [19] and new polymers with increased intrinsic microporosity [20]. 

However, the acceptance of membrane technology continues being hindered by the materials’ stability under operating conditions. In order to account for the uncertainty of the membrane behavior in the presence of impurities [21], Chenar et al. [22] compared two commercial polymer hollow fiber membranes with different hydrophilic (Cardo polyimide) and hydrophobic (PPO) selective layer in dry and wet conditions. They observed that the CO_2_ and CH_4_ permeances were differently affected by the hydrophobic or hydrophilic character of the membranes in CO_2_/CH_4_ separation in the presence of water vapor in the feed. 

Simcik et al. observed that the water affinity has a key role for the separation of CO_2_/CH_4_ as representative biogas binary mixture [23]. Likewise, the permeance and selectivity of glassy polyimide membranes have been affected by the presence of wet non-methane hydrocarbons in natural gas applications. For example, the CO_2_ permeance and CO_2_/CH_4_ selectivity of 6FDA-based dense membranes and CA thin-film composite membranes were observed to decrease in the presence of n-hexane or pentane in the feed gas, due to the plasticization of the polymer [6,24]. On the other hand, the presence of toluene caused a decrease of CO_2_ permeance of cross-linkable polyimide membranes [25,26] attributed to competitive sorption of toluene impurity. Cerveira et al. [27] compared two different polymer membranes, cellulose acetate (CA) and polydimethylsiloxane (PDMS), in the separation of CO_2_ from CH_4_ at 22 °C and 50 psig, observing that CO_2_ caused plasticization, resulting in lower separation factors in the mixed-gas mixture separation tests that were accentuated by the presence of impurities other than water vapor in the feed.

Hydrophilicity, as the other membrane properties, like mechanical resistance, pore size and morphology, can be tuned up by modifying membrane material composition [28]. The hydrophobic or hydrophilic character of a membrane may help resolve mass transfer limitations and the removal of impurities in the biogas feed that prevent the integration of membrane technology in biogas production plants [8,29]. Hydrophilicity can influence CO_2_ facilitated transport in mixed matrix membranes by modification of “Janus” materials with Ag^+^ ions [30]. In a recent work, we observed that the hydrophobicity of PTMSP can be changed by adding hydrophilic Zeolite 4A to polymer matrix, obtaining a new mixed matrix membrane material with increasing CO_2_ permeance and increasing or constant selectivity with increasing relative humidity in the feed up to 50% [31]. However, when reducing the thickness of the membranes to prepare thin-film composite membranes, the high selectivity of this MMM was significantly decreased [32] as the probability of defects grew, as usual in MMM synthesis [33]. This was not observed when the chitosan biopolymer (CS) matrix hybridized with 1-ethyl-3-methylimidazolium acetate ([emim][acetate]) ionic liquid (IL) as filler was coated on the polyethersulfone (PES) support, because of the good compatibility between IL and CS [34]. Surface modification of robust supports is a common way of tuning up the membrane separation properties and correcting defects [35].

The aim of this study was to investigate the performance of two types of highly CO_2_ high permeable thin-film composite membranes whose selective layer possesses different hydrophobic and hydrophilic character, in the separation of CO_2_/CH_4_, in the presence and absence of water vapor and organic pollutants. These membranes were previously studied in the separation of CO_2_/N_2_ gas mixtures [32,36], so the purpose of the present work is to estimate the potential of membranes for biogas upgrading. 

## 2. Materials and Methods

### 2.1. Materials and Modules

The membranes used for this study are flat-sheet composite Membranes, both PDMS PERVAP 4060 (Sulzer Chemtech GmbH, Alschwill, Switzerland), with a 1–1.5 μm thick PDMS top layer and a total thickness of 180 μm, and the IL–CS/PES composite membrane fabricated in our laboratory, with similar selective layer thickness of about 1.5 μm as the commercial hydrophobic membrane to facilitate comparison [32].

### 2.2. Gas Permeation Experiments

**Figure 1:**
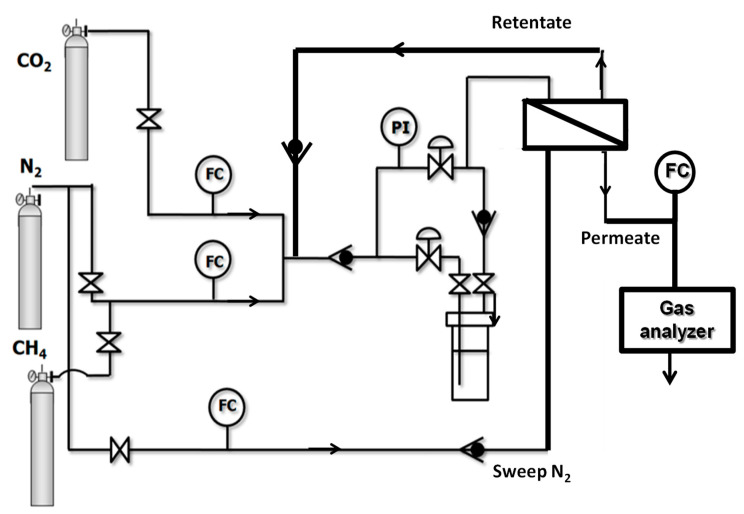
Diagram of the gas separation plant used in the experiments.

The flat composite membranes studied in this work, with an effective membrane area of 15.6 cm^2^, were placed in a stainless-steel module and tested in the separation of CO_2_/CH_4_ as a function of feed composition in a homemade separation plant represented in Figure 1, which enables working with different module geometry configuration in dry and humid feed conditions [37]. The feed gas concentration was set by mass flow controllers (KOFLOC 8500, Sequopro S.L., Madrid, Spain), while the retentate and permeate flow rates were measured by a bubble flow meter at the exit of the permeate. Flow control was achieved by means of backpressure regulators on the retentate stream. Permeate was kept at atmospheric pressure, and N_2_ was used as sweep carrier gas at a flow rate 10 mL/min. 

Once the membrane performance reached the steady state, the permeate was measured by using a bubble flow meter at the end of the system, at least 3 times, for about 1 h, to confirm the membrane stability at a given operating condition. The composition of the permeate stream is determined by a gas analyzer (BIOGAS5000, Geotech, Tamarac, FL, USA).

As reported in our previous work with composite hollow fiber membranes [37], the humid gas experiments were carried out at approximately 50% relative humidity in the feed by passing half of the feed gas stream through a tank filled with water before entering the module. Stop-valves prevented the entrance of liquid water into the membrane modules. Once the performance of the membranes has been characterized in dry and humid conditions, 2 mL toluene (Sigma Aldrich, St. Louis, MI, USA) was added, as a model organic contaminant [26] to the water tank, and the humid experiments were repeated in the presence of this organic contaminant, for both types of membranes. 

The permeance of gas *i*, (*P*/*t*)_*i*_, in GPU (1 GPU = 10^−6^ cm^3^(STP) cm^−2^ s^−1^ cmHg^−1^), is defined as the pressure-normalized flux of a gas through a membrane:

(1) $${\left(\frac{P}{t}\right)}_{i}=\frac{Q_{p}y_{i}}{\left(p_{r}x_{i}-p_{p}y_{i}\right)A}\times 10^{6}$$

where *P* is the intrinsic permeability of the selective membrane layer, in Barrer (1 Barrer = 10^−10^ cm^3^(STP) cms^−1^ cmHg^−1^); *p*_*p*_ is the retentate and permeate pressure, in cmHg, respectively; *A* the effective area of the membrane; *t* is the selective layer thickness for the separation; and *Q*_*p*_ is the permeate flow rate (cm^3^ (STP)/s) at measurement pressure and temperature conditions.

The separation factor of gas *i* over gas *j*, α_*ij*_, is defined as the ratio of the concentration of gas *i* in the permeate and the retentate relative to the same ratio for gas *j*:

(2) $$\alpha_{ij}=\frac{\left(y_{i}/y_{j}\right)}{\left(x_{i}/x_{j}\right)}$$

The selectivity is calculated as the ratio between the permeance of the fast and slow gas components in a gas pair, in the case of this work, CO_2_ and CH_4_, respectively.

## 3. Results

### 3.1. Membrane Performance in Terms of the Robeson’s Upper Bound

The most popular means of comparing membrane performances for gas pair separations acknowledged worldwide is the Robeson’s plot, usually on the development of new membrane materials [17]. Although initially settled from pure gas permeances and selectivities, this upper bound was also employed to analyze the effect of mixed gas feed mixtures in the separation performance of available or new membranes. This upper bound has been redefined by Professor Robeson and other authors, in order to take new developments on membrane materials and configurations into account [20]. 

**Figure 2:**
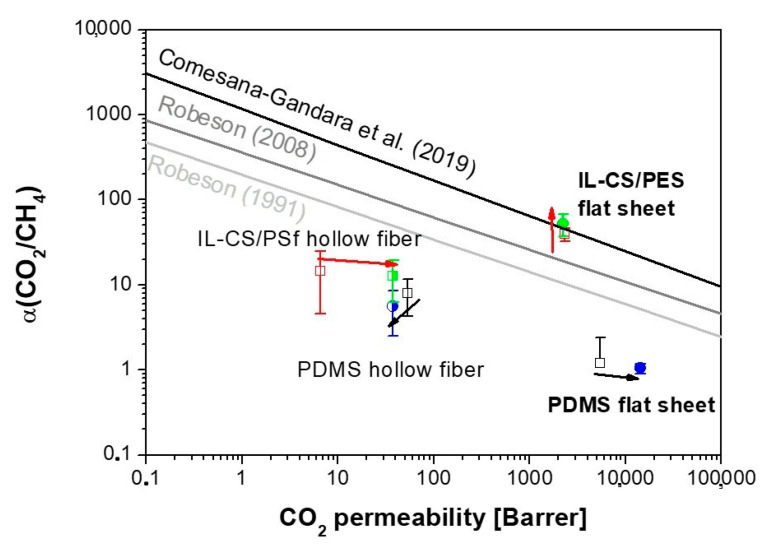
Effect of morphology on the CO_2_/CH_4_ separation performance of the fresh hydrophobic PDMS and hydrophilic ionic liquid–chitosan (IL–CS) composite membranes, against the CO_2_/CH_4_ upper bound.

The upper bound developed by Robeson and the modifications for new polymers upgraded [20] are also plotted for comparison with the pure gas upper bound Robeson’s plot in Figure 2 [17]. This upper bound is an useful screening tool for the development of new membrane materials, usually as self-standing single-layer membranes, but it has limitations to establish the state-of-the-art of thin-film composite membranes as those commercially available, since the trade-off plots described are for pure-gas permeation [38]. 

In this light, for instance, Lin and Yavari highlighted that the upper bound for CO_2_/CH_4_ mixed-gas separation in the presence of non-methane hydrocarbon impurities could reduce the upper bound up to 37% for 20%CO_2_/80%CH_4_ mixtures [21]. They developed a model based on the free volume theory, to define the variations observed for composite membranes and process operation variables (such as pressure or the presence of contaminants) when compared with the upper bound generated from single-gas permeation depicted in Figure 2. The upper bound has been revised to include high free-volume polymers with intrinsic porosity [39], which intrinsically give much higher permeabilities and lower selectivities than other polymers, thus making the comparison difficult. These high free-volume polymers are also susceptible to suffer from phenomenological effects such as plasticization and physical aging, in detrition of their CO_2_ separation performance. Specifically, Comesaña-Gandara et al. revised the CO_2_/CH_4_ upper bound, in order to include novel polymers whose intrinsic microporosity provided high permeability and selectivity not contemplated in previous upper bounds [20]. This upper bound is utilized in Figure 2, to compare the influence of morphology when comparing dense- and thin-film composite IL–CS/PES membranes reported elsewhere for CO_2_/N_2_, regarding their CO_2_/CH_4_ separation performance. The performance is observed to be highly dependent on the active layer thickness, increasing up to the latter upper bound mentioned. Those authors studied polyimide membranes with the same thickness and flat-sheet configuration we used in this work. In this work, we compare the performance of this hydrophilic IL–CS/PES and hydrophobic commercial PDMS membrane in the separation of CO_2_/CH_4_ mixtures, in the presence of humidity and organic pollutants. 

### 3.2. Comparison of Membrane Performance in the Presence of Impurities

The permeability and selectivity in Figure 2 were calculated from single-gas permeation experiments as a function of feed pressure, between 2 and 5 bar. The feed pressure in biogas pilot plant experiments is not usually very high, so the gas-mixture separation experiments were performed at a feed pressure of about 4.5 bar, focusing on the influence of other components in the mixture that could affect the gas-separation performance by the presence of impurities in the feed, upon feed concentration and flux decline by membrane plasticization, as well as decrease of glass transition upon addition of organic pollutants [40]. 

**Figure 3:**
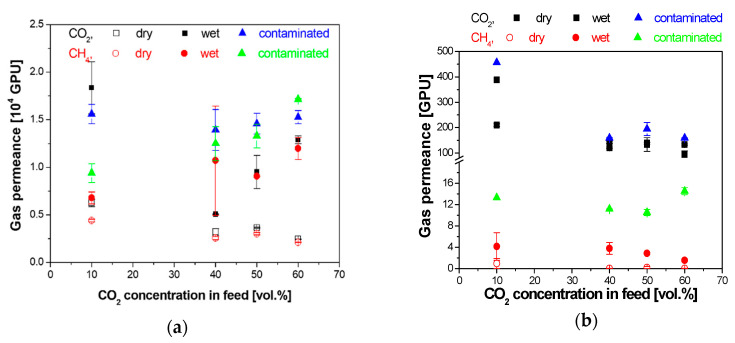
Influence of dry (void symbols) and humid (full symbols) conditions of the feed in the CO_2_ (black squares) and CH_4_ (red circles) permeance through the hydrophobic PDMS (**a**) and the hydrophilic IL–CS flat composite membrane (**b**). Data obtained after contamination with the toluene solution are drawn by the full triangles (blue for CO_2_, and green for CH_4_).

The experimental results of the influence of water vapor in the feed on the CO_2_ and CH_4_ permeances obtained in this work are represented in Figure 3 for a flat hydrophobic and a hydrophilic composite membrane, respectively. The CO_2_ and CH_4_ dry permeances through the IL–CS composite membranes are of the same order of magnitude as those reported for a commercial CA membrane [41]. The results shown in Figure 3 for the CO_2_:CH_4_ separation in the presence of humidity affect the hydrophilic IL–CS/PES composite membrane differently than the PDMS membrane. The presence of toluene as model organic pollutant in the humid feed stream enhances these differences. The permeance of the hydrophobic membrane is affected by the presence of damp impurities, while the permeance and selectivity of the hydrophilic membrane are almost invariable in the presence of humid streams.

As observed in composite hollow fiber membrane geometry elsewhere, the presence of water vapor in the feed affects most significantly the CO_2_ permeance through the hydrophobic PDMS than the hydrophilic IL–CS/PES membrane [22,37]. The permeances through the hydrophobic membrane, in Figure 3a, are not as affected by the CO_2_ concentration in the feed in the whole range of feed gas concentration as by the humidity or the presence of organic pollutant, while the behavior of the hydrophilic membrane (Figure 3b) is mostly affected by CO_2_ concentration in the feed rather than the presence of impurities. The CO_2_ permeances decrease with increasing CO_2_ concentration in the feed in a more remarkable way for the hydrophobic PDMS composite membrane than for the hydrophilic IL–CS-based composite membrane, which may be attributed to the water-facilitated transport through the hydrophilic membrane [14].

NaOH treatment is used in IL–CS-based membranes to neutralize the acetate groups of the CS polymer matrix and enhance the affinity towards acid gas molecules such as CO_2_. This treatment has also been used to increase CO_2_ separation properties from other gases in ZrO_2_ ceramic membranes that were not so selective as-made [42]. NaOH immersion in MMMs has also been reported to enhance the hydrophilicity and facilitated transport through Ag^+^-particles MMMs in CO_2_/CH_4_ separation [30]. In chitosan-based membranes, the same NaOH treatment is used to neutralize the functional groups of the IL–CS matrix to attract CO_2_ preferentially [43]. After immersion in NaOH 1M and removal of excess NaOH solution from the surface of the membrane, the membrane is dried at 50 °C prior to characterization. The selectivity of the hydrophobic PDMS commercial membrane increases both in single-gas permeation and gas-mixture separation experiments after this treatment. Table 1 collects these characterization results for the case of dry feed gas measurements. Although the CO_2_ permeance of the PDMS membrane decreases in the order of magnitude 86–95% upon NaOH treatment, the permeance of the NaOH-treated PDMS membrane is 169 ± 9.9 GPU, 165 ± 10 GPU and 320 ± 39 GPU for 60/40, 50/50 and 40/60 CO_2_/CH_4_ (vol %) gas mixture concentrations in the feed. These values are in the order of magnitude reported in the literature [26]. The CH_4_ permeance decreases more than 99% and determines the enhancement of the CO_2_/CH_4_ selectivity of the membrane summarized for the dry membranes in Table 1.

The relationship between the permeable gas and the gas pair selectivity obtained for the NaOH-treated membranes follows the same trend that was reported by Lokhandwala et al. [41] for N_2_/CH_4_ separation.

**Figure 4:**
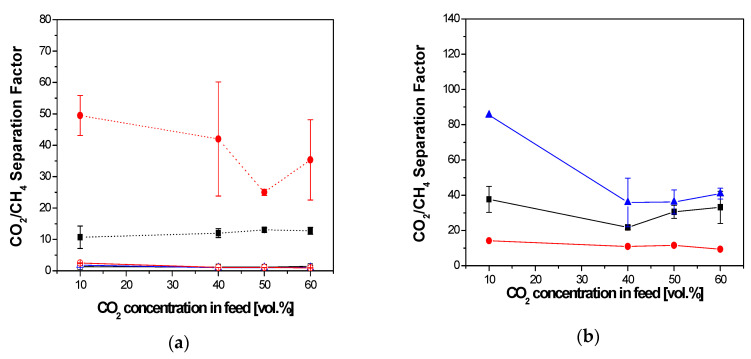
Influence of feed conditions on the CO_2_/CH_4_ separation factor of the commercial hydrophobic PDMS membrane (**a**) and the hydrophilic IL–CS/PES (**b**), in the absence (squares) and presence of water vapor (blue) and toluene (red) impurities. Void symbols in Figure 4a correspond to the experimental values measured with the un-treated commercial PDMS membrane for comparison purposes. Lines are a guide to the eye.

The separation factor through the membranes is plotted in Figure 4. The hydrophobic PDMS membrane shows lower CO_2_/CH_4_ selectivity than the hydrophilic IL–CS composite membrane (Figure 4). The CO_2_/CH_4_ of the untreated hydrophobic PDMS membrane is not affected by feed-gas-mixture concentration. This agrees with the study of Chenar et al., when they compared the performance of Cardo polyimide with PPO hollow fiber membranes in dry and humid CO_2_/CH_4_ gas mixture separation [22]. The CO_2_/CH_4_ separation factor of the PDMS membrane was increased by the NaOH-treatment for the whole range of CO_2_ concentration in the feed mixture. As expected, the CO_2_/CH_4_ separation of the hydrophilic membranes was not diminished by the presence of water vapor, as it was also previouslyobserved in Figure 3b.

The red full circles in Figure 4 represent the influence of the presence of toluene contaminating the feed to the membrane module. This corresponds to a toluene concentration of 3460 ppm, which is high enough to accelerate the observation of any contamination effect affecting the aging of the membrane-separation operation in a single experiment [25]. The decrease of CH_4_ permeance through the rubbery hydrophobic membranes with increasing CO_2_ concentration in the feed was not significant due to the competition between sorption and plasticization of the CO_2_ in the PDMS layer. In the case of the hydrophilic IL–CS/PES membrane, there was a favorable competition between plasticization and water preferential solubility for CO_2_ [19]. This agrees with the favorable competition on CO_2_ and CH_4_ permeances reported by Jusoh et al. for polyamide membranes in the presence and absence of humid pentane [6].

On the other hand, Figure 4 shows how the presence of the model organic pollutant, toluene, in the feed mixture increased the separation factor of the rubbery hydrophobic PDMS membrane, but decreased that of the hydrophilic IL–CS/PES membrane. This may be compared to the antiplasticizing effect of toluene in SSZ-13-PDMC mixed matrix membranes (hydrophobic) and un-crosslinked Matrimid membranes (more hydrophilic) observed by professor Koros’ group [25,26]. The comparatively large increase in selectivity of the NaOH-treated PDMS membrane may be due to the organic pollutant blocking the pass of the CO_2_ and CH_4_ molecules through the membrane [25]. Since this was particularly remarkable for the NaOH-treated PDMS membrane, one may point out to some changes in the chemical composition of the membrane surface altering the interaction of the membrane surface with the gas feed mixture and thus the CO_2_/CH_4_ separation performance. 

### 3.3. Surface Characterization

**Figure 5:**
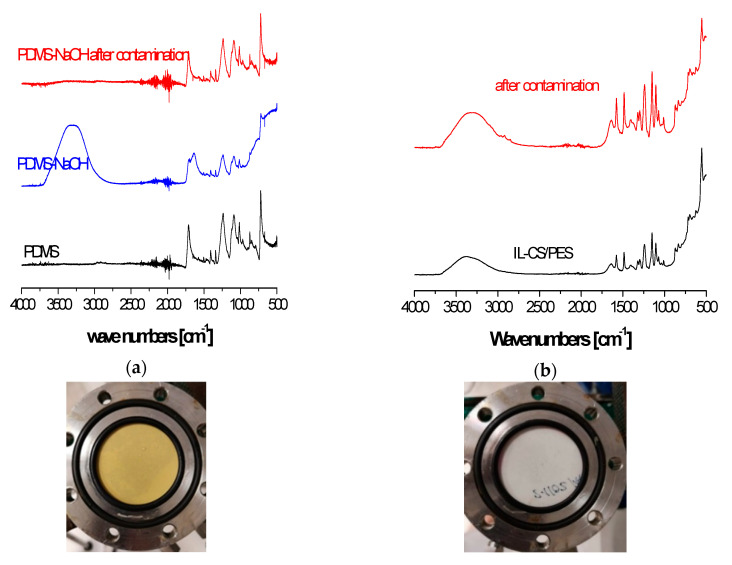
ATR–FTIR spectra of the PDMS (**a**) and IL–CS/PES (**b**) membranes. Pictures of the top surface of the composite membranes after 10 h experimental runs are presented below.

ATR–FTIR has been used to characterize the surface chemistry of fresh membranes before their separation performance [44]. In this work, the ATR–FTIR spectra in Figure 5a reveal that the NaOH treatment increases the hydrophilicity of the PDMS hydrophobic layer, as derived by the band at 3500 cm^−1^, typical of the OH stretching vibration, appearing at the NaOH-treated PDMS surface, measured on the dry membrane. This accounts for the different performance of the PDMS membrane after an NaOH treatment. The PDMS characteristic peaks at 2964–2950 cm^−1^ are still present after NaOH treatment, so the main polymer backbone of the top layer of the commercial membrane should not have been damaged after the experimental runs in the presence of toluene in the feed gas mixture, although the magnitude of the OH band of the freshly NaOH-treated PDMS membrane makes it difficult to discern these peaks in the clean diagram [45]. 

Regarding the effect of the organic contaminant, the 1024 and 1070 cm^−1^ bands of in-plane C–H stretching of toluene are appreciated in the contaminated IL–CS/PES membrane in Figure 5b. These bands are blurred in the PDMS-based membranes spectra in Figure 4a before contamination, so no conclusion can be extracted at this point. No other apparent changes are appreciated in the IL–CS IR peaks [46] in the IL–CS/PES membrane. This implies that the membrane is not deteriorated by the presence of toluene contaminant, which agrees with the different coloring of the membrane surface of the hydrophobic (left) and hydrophilic (right) membranes included in the pictures below the spectra. The PDMS-based membrane from Sulzer was also reported to turn yellowish brown, as well after CO_2_ separation from flue gas in pilot plant measurements [47].

## 4. Conclusions

Biogas upgrading development is one of the key features to attain circular economy. The main drawback of membrane technology to be actually seen as the potential technical and environmentally friendly alternatives on the treatment of residual gas effluents is the uncertainty of available membranes’ behavior in the presence of impurities in practical waste feed stream mixtures. This work aims at analyzing the behavior of membranes in the presence and absence of water and model organic compounds as a function of the membrane’s top-layer characteristics. The perm-selectivity of commercial PDMS membranes can be altered by an alkaline treatment commonly used to stabilize polyelectrolyte membranes commonly studied as ion-exchange membranes in several applications, including acid gas purification. 

This NaOH treatment facilitating the transport properties, i.e. the CO_2_ permeance and CO_2_/CH_4_ separation characteristics of the hydrophilic IL-CS/PES membrane even in the presence of impurities, also maintains the transport properties, of the hydrophobic PDMS membrane regardless the CO_2_ concentration in the feed.

The tuning up of the hydrophilic/hydrophobic character of the membrane surface can be an effective way of improving facilitated transport properties and improving membrane performance in CO_2_ capture applications. The outcomes of this study are being validated by gas separation models, in order to analyze the correlation between the presence of impurities and the membrane performance as an approach to overcome the uncertainty of membrane technology in the treatment of residual gas streams. 

## Figures and Tables

**Table 1:** Theoretical and mixed gas selectivity results for hydrophobic PDMS and hydrophilic IL–CS/polyethersulfone (PES) membranes.

Membrane	Theoretical ^1^ α(CO_2_/CH_4_)	Mixed Gas CO_2_/CH_4_ Selectivity		
		60/40 (vol %)	50/50 (vol %)	40/60 (vol %)
PDMS	3.13	1.15	1.21	1.26
IL–CS/PES	2.66	48.47 ± 8.6	39.59 ± 7.0	36.49 ± 2.6
PDMS–NaOH	10.65	16.52 ± 2.0	15.0 ± 0.3	12.11 ± 1.42

^1^ From single gas-permeation experiments.
